# Supplementary material for: A Systematic Review and Meta-Analysis on the Efficacy of Locally Delivered Adjunctive Curcumin (Curcuma longa L.) in the Treatment of Periodontitis
Source: Biomedicines. 2023 Feb 7;11(2):481. doi: 10.3390/biomedicines11020481 (PMC9953093; doi:10.3390/biomedicines11020481)
Supplement: Supplementary file 1 [file biomedicines-11-00481-s001.zip › biomedicines-2127529-supplementary.pdf]

Supplementary Material

Table S1. Search strategies for each database and search results.

**1.Pubmed**		
**Search number**	Search	Results
**1**	"Periodontitis"[Mesh] OR "Periodontal Diseases" [Mesh:NoExp]	56,466
**2**	periodontitis[Title/Abstract] OR periodontal[Title/Abstract]	78,106
**3**	#1 OR #2	94,321
**4**	"Curcuma"[Mesh] OR "Curcumin"[Mesh] OR "turmeric extract" [Supplementary Concept]	14,607
**5**	turmeric[Title/Abstract] OR curcumin[Title/Abstract]	19,415
**6**	#4 OR #5	20,957
**7**	#3 AND #6	158
**8**	#7 NOT ("Animals"[Mesh] NOT "Humans"[Mesh])	133
**2.Cochrane Library (Wiley)**		
**Search number**	Search	Results
**#1**	[mh Periodontitis] OR [mh ^"Periodontal Diseases"]	3667
**#2**	periodontitis:ti,ab,kw OR periodontal:ti,ab,kw	12206
**#3**	#1 OR #2	12243
**#4**	[mh Curcuma] OR [mh Curcumin]	567
**#5**	turmeric:ti,ab,kw OR curcumin:ti,ab,kw OR curcuma:ti,ab,kw	1913

**#6**	#4 OR #5	1913
**#7**	#3 AND #6	79
**3. BASE (base-search.net)**		
	Search	Results
	(periodontitis periodontal) AND (turmeric curcumin)	305
**4. ClinicalTrial.gov**		
	Search	Results
	Condition: periodontitis OR periodontal | <br>Intervention: turmeric OR curcumin OR curcuma	7
**5. LIVIVO**	Search	Results
	(periodontitis OR periodontal) AND (turmeric OR curcumin)	191
**6. Dentistry Oral Sciences Source (Ebsco) and MEDLINE Complete (Ebsco)**	Search	Results
	(periodontal OR periodontitis) AND (curcumin OR turmeric OR curcuma) AND (randomized OR rtc OR randomised)	55
**7. Scopus.com (Elsevier)**	Search	Results
	TITLE-ABS-KEY ((periodontitis OR periodontal) AND (turmeric OR curcumin) AND random*)	54
